# Hyperglycemia Modulates mTOR Signaling and Myelin Protein Expression in Schwann Cells

**DOI:** 10.3390/ijms26199724

**Published:** 2025-10-06

**Authors:** Nurul Husna Abd Razak, Ubashini Vijakumaran, Izyan Mohd Idris, Jalilah Idris, Nur Hidayah Hassan, Fazlin Zaini, Noorzaid Muhamad, Muhammad Fauzi Daud

**Affiliations:** 1 Institute of Medical Science Technology, Universiti Kuala Lumpur (UniKL), A1-1, Jalan TKS 1, Taman Kajang Sentral, Kajang 43000, Selangor, Malaysia; husna.razak01@s.unikl.edu.my (N.H.A.R.); ubashini@unikl.edu.my (U.V.); jalilahidris@unikl.edu.my (J.I.); nurhidayah@unikl.edu.my (N.H.H.); 2 Institute for Medical Research (IMR), National Institutes of Health (NIH), Ministry of Health Malaysia, Jalan Setia Murni U13/52, Seksyen U13, Setia Alam, Shah Alam 40170, Selangor, Malaysia; izyan.idris@imr.gov.my; 3 Royal College of Medicine Perak, Universiti Kuala Lumpur (UniKL), No. 3, Jalan Greentown, Ipoh 30450, Perak, Malaysia; fazlin@unikl.edu.my (F.Z.); noorzaid@unikl.edu.my (N.M.)

**Keywords:** diabetic peripheral neuropathy, Schwann cell reprogramming, hyperglycemia, plasticity, mTOR signalling

## Abstract

Diabetic peripheral neuropathy (DPN) is a common complication of diabetes, marked by Schwann cell dysfunction, demyelination, and impaired nerve regeneration. Although Schwann cells undergo phenotypic changes under hyperglycemic conditions, the underlying molecular mechanisms remain unclear. This study aimed to examine the effects of high glucose on Schwann cell phenotype and assess the involvement of the mTOR signaling pathway. Primary Schwann cells were isolated from rat sciatic nerves and cultured in media containing 5 mM (control), 25 mM, or 50 mM glucose for five days. Immunofluorescence staining and corrected total cell fluorescence (CTCF) analysis were used to evaluate expression of key markers: c-Jun, Krox-20, p75^NTR^, MBP, mTOR, phosphorylated mTOR (Ser2448), and AKR1B1. Among these, significant changes were observed in MBP (*p* = 0.002), total mTOR (*p* = 0.001), and phosphorylated mTOR (Ser2448) (*p* = 0.0179), indicating impaired mTOR activation and loss of myelin protein expression. Non-significant changes in the other markers are discussed as preliminary observations. These findings highlight mTOR dysregulation and impaired myelin protein expression as central features of Schwann cell responses to hyperglycemia, which may contribute to the development of DPN.

## 1. Introduction

Diabetic peripheral neuropathy (DPN) is one of the most prevalent and severe complications of diabetes, affecting a large proportion of individuals living with the disease [1]. It can result in a range of debilitating outcomes, from early symptoms like paresthesia to more severe complications such as limb loss and even death. Research indicates that between 10% and 20% of diabetic patients are diagnosed with DPN at the time of their diabetes diagnosis, and an estimated 50% to 66% of patients with diabetes will develop DPN over their lifetime [2,3]. High blood glucose levels have been identified as a major contributing factor, driving oxidative stress, chronic inflammation, and cellular damage, which all play a role in the development and progression of DPN [4]. Although DPN is widespread, its exact cause remains elusive [5]. The pathophysiology of DPN involves multiple interconnected processes, including axonal atrophy, Schwann cell demyelination, and a loss of nerve regenerative capacity. Schwann cells form the myelin sheath around axons, allowing efficient nerve signal transmission [6]. However, in diabetic patients, Schwann cells show signs of dedifferentiation and demyelination, impairing their function and hindering the ability to repair damaged nerves [7,8]. This leads to the progressive loss of nerve function and exacerbates the clinical symptoms of DPN, such as sensory loss, muscle weakness, and discomfort [9]. Several hypotheses have been proposed to explain the molecular and cellular mechanisms underlying DPN, with metabolic dysfunction [10], microvascular damage [11], and inflammatory dysregulation [12] being some of the leading theories.

Meanwhile, high blood glucose levels contribute to a toxic environment in peripheral nerves by inducing oxidative stress [13]. This oxidative stress disrupts critical cellular signaling pathways, thereby impairing Schwann cell function and diminishing the regenerative potential of peripheral nerves [14]. Among these disrupted pathways, the polyol and poly(ADP-ribose) polymerase (PARP) pathways have been extensively studied in the context of diabetic neuropathy. In contrast, pathways such as Wnt, mitogen-activated protein kinase (MAPK), and the mechanistic target of rapamycin (mTOR) have more recently emerged as important contributors to Schwann cell dysfunction and nerve degeneration under hyperglycemic conditions [15]. Under normal conditions, mTOR supports Schwann cell function by controlling cellular processes such as growth, differentiation, and myelin formation. However, in hyperglycemia, dysregulated mTOR activation contributes to Schwann cell plasticity, mitochondrial dysfunction, and dedifferentiation [16]. In Schwann cell injury response, c-Jun expression was induced by the temporary activation and upregulation of the mTORC1 signaling pathway [17], suggesting a shift toward a repair phenotype [18]. Meanwhile, Krox-20 expression, which is essential for myelination, downregulated in a repair phenotype that favors dedifferentiation and remyelination inhibition [19]. Aldose reductase, Aldo-keto reductase family 1 member B1 (AKR1B1), is also a highly expressed protein in Schwann cells that catalyzes the conversion of glucose to sorbitol and ultimately to fructose by sorbitol dehydrogenase [20]. Their activation and accumulation of sorbitol in Schwann cells under hyperglycemia play a key role in the pathogenesis and progression of DPN [20,21].

Our previous study demonstrated that in vitro cultured Schwann cells do not dedifferentiate into an immature phenotype, as traditionally thought, but rather reprogram into a distinct repair phenotype characterized by elevated c-Jun and p75^NTR^ expression and reduced Krox-20 and MBP levels [7]. Building on these findings, the current study aims to further explore the molecular mechanisms driving Schwann cell plasticity under diabetic conditions by focusing on the regulatory roles of mTOR activation, c-Jun, Krox-20, and AKR1B1 in Schwann cell reprogramming during hyperglycemia.

## 2. Results

### 2.1. Immunofluorescence Analysis on the Expression of c-Jun and Krox-20, Transcription Factors That Regulate Schwann Cell Phenotypes

**Figure 1:**
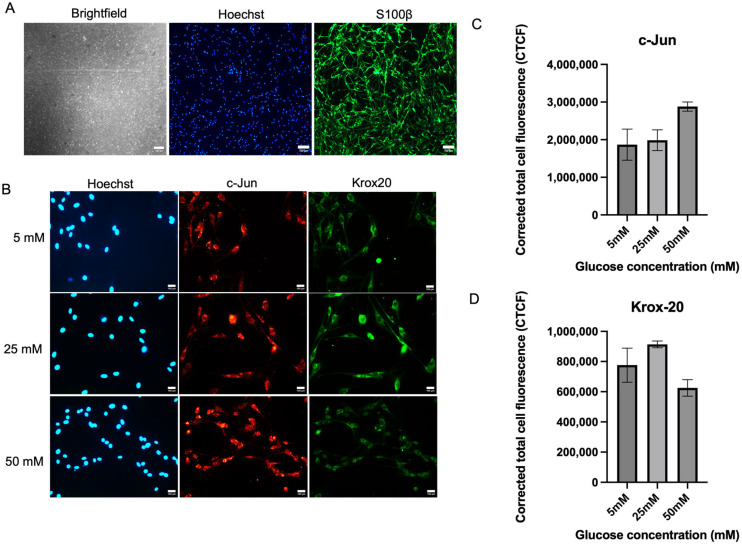
Expression of c-Jun and Krox20 in Schwann cells. (**A**) Brightfield picture of Schwann cell culture and its positive expression of S100β. (**B**) Representative immunofluorescence micrographs of Schwann cells expressing c-Jun and Krox20 under 40× magnification. Schwann cells have been cultured in 5 mM, 25 mM, and 50 mM of glucose for 5 days and were stained with Hoechst, c-Jun, and Krox-20. Scale bar = 100 µM. (**C)** Measurement of corrected total cell fluorescence for repair transcription factor, c-Jun expression, (**D**) Measurement of corrected total cell fluorescence for myelinating transcription factor Krox-20 expression. The mean CTCF value was presented as mean ± SD. (*n* = 3, three independent experiments).

Figure 1A shows an in vitro culture of Schwann cells with positive expression of S100β. (Figure 1B–D) Schwann cell culture expressed an increased trend of c-Jun expression with no significant differences between the three glucose concentration groups. c-Jun expression was the highest in high glucose media (50 mM) with a mean of 2,881,296 ± 121,760 compared to 5 mM (1,867,655 ± 413,012), *p* = 0.1124, and 25 mM (1,984,390 ± 277,093), *p* = 0.9582. Meanwhile, Krox-20 expression in the 50 mM media was the lowest, with no significant differences detected as shown in Figure 1D. The mean value of Krox-20 expression in 50 mM media was (625,218 ± 54,618) compared to 5 mM (776,103 ± 112,833), *p* = 0.3763 and 25 mM (913,606 ± 22,652), *p* = 0.4348. High glucose exposure showed a non-significant trend toward increased c-Jun and decreased Krox-20 expression, changes that may suggest early features of a repair-like phenotype but did not reach statistical significance.

### 2.2. Immunofluorescence Analysis on the Expression of p75^NTR^ and MBP in In Vitro Cultured Schwann Cells

**Figure 2:**
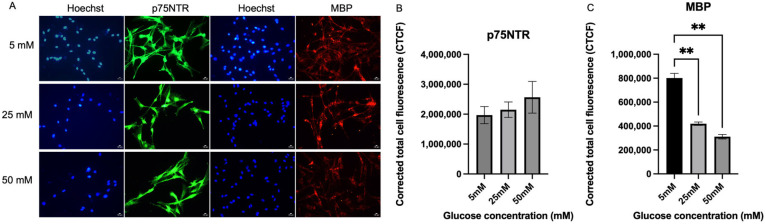
Expression of p75^NTR^ and MBP. (**A**) Representative immunofluorescence micrographs of Schwann cells expressing p75^NTR^ and MBP under 40× magnification. Scale bar = 50 µm. Schwann cells have been cultured in 5 mM, 25 mM, and 50 mM of glucose for 5 days and stained for p75^NTR^ and MBP. (**B**) Measurement of corrected total cell fluorescence for p75^NTR^ in three different culture media (5 mM, 25 mM, 50 mM). (**C**) Measurement of corrected total cell fluorescence for MBP in three different culture media (5 mM, 25 mM, 50 mM). The mean CTCF value was presented as mean ± SD, ** *p* < 0.001. (*n* = 3, three independent experiments).

Figure 2A shows the expression of p75^NTR^ and MBP of Schwann cells in hyperglycemic conditions. For p75^NTR^, there were no significant differences between the culture media with three different glucose concentrations. The expression level of p75^NTR^ was expressed highest by Schwann cells in 50 mM culture media (2,564,124 ± 529,524) when compared to 25 mM (2,149,244 ± 258,103), *p* = 0.7306 by 19.35% and 5 mM (1,967,167 ± 287,152), *p* = 0.5399 by 30.35%, but no significant difference was detected. However, Schwann cells in the 5 mM media showed the highest MBP expression (801,545 ± 391,903) compared to 25 mM media (419,577 ± 14,520), with a significant difference (*p* = 0.0041) and an 81.6% increase, and 50 mM media (310,960 ± 18,573), with a significant difference (*p* = 0.0020) and a 157.8% increase.

### 2.3. Immunofluorescence Analysis on the Expression of Aldose Reductase in In Vitro Cultured Schwann Cells

**Figure 3:**
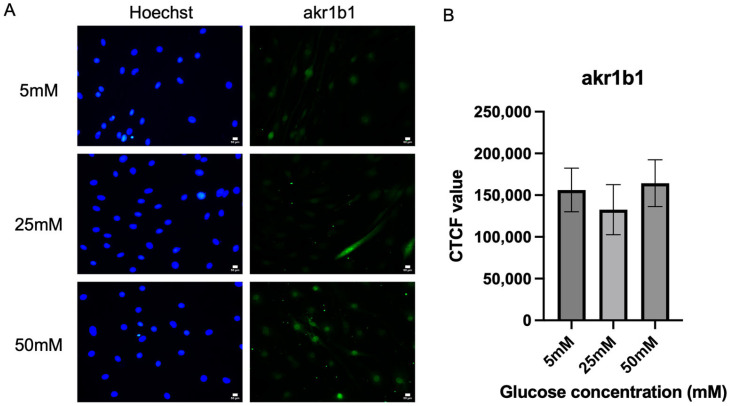
Expression of akr1b1. (**A**) Representative immunofluorescence micrographs of Schwann cells expressing akr1b1 under 40× magnification. Scale bar = 50 µm. Schwann cells have been cultured in 5 mM, 25 mM, and 50 mM of glucose for 5 days and stained for akr1b1. (**B**) Measurement of corrected total cell fluorescence for aldose reductase in three different culture media (5 mM, 25 mM, 50 mM). The mean CTCF value was presented as mean ± SD. (*n* = 3, three independent experiments).

Figure 3A shows the representative fluorescence micrograph images of Schwann cells expressing aldose reductase (akr1b1), which is the key enzyme for the polyol pathway across all three different glucose concentration media (5 mM, 25 mM, and 50 mM). The aldose reductase expressions were detected within the nucleus of the cells. There is no significant difference in akrb1 expression across the tested hyperglycemia conditions. Figure 3B shows that there were no significant differences between the expression of aldose reductase (akr1b1) in Schwann cells cultured in three different glucose media. However, expression of aldose reductase was seen as highest in the 50 mM glucose media (164,315 ± 27,988) when compared to 25 mM media (132,633 ± 29,987), *p* = 0.8288, and 5 mM glucose media (156,192 ± 26,158), *p* = 0.9773. Expression of aldose reductase (akr1b1) by Schwann cells cultured in 50 mM glucose media was 23.89% higher when compared to the Schwann cells cultured in 25 mM glucose media. A slight but non-significant increase in AKR1B1 expression was observed in Schwann cells exposed to high glucose, which may indicate a potential trend toward polyol pathway involvement.

### 2.4. Immunofluorescence Analysis on the Expression of mTOR In Vitro Cultured Schwann Cells

**Figure 4:**
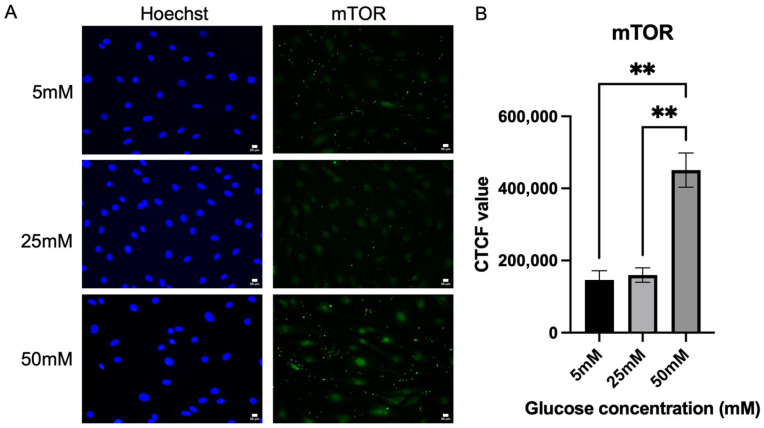
Expression of mTOR. (**A**) Representative immunofluorescence micrographs of Schwann cells expressing mTOR under 40× magnification. Scale bar = 50 µm. Schwann cells have been cultured in 5 mM, 25 mM, and 50 mM of glucose for 5 days and stained for mTOR. (**B**) Corrected total cell fluorescence for mTOR in three different culture media (5 mM, 25 mM, 50 mM). The fluorescence intensity of mTOR was quantified to observe the effect of hyperglycemia on the mTOR activity. The mean CTCF value was presented as mean ± SD, ** *p* < 0.001. (*n* = 3, three independent experiments).

Following the investigation of the activation of the polyol pathway, this study also examined the expression level of mTOR. mTOR was also quantified by measuring the fluorescence intensity, as shown in Figure 4. A significant increase in mTOR expression was detected in the Schwann cells cultured in 50 mM glucose concentration media (450,901 ± 47,517), *p* = 0.0015 when compared to Schwann cells cultured in 5 mM (146,390 ± 25,282) and 25 mM glucose concentration media (159,830 ± 20,053). Schwann cells cultured in 50 mM glucose media expressed mTOR by 182.113%, significantly higher than 25 mM glucose media.

### 2.5. Immunofluorescence Analysis on the Expression of Phosphorylated mTOR in In Vitro Cultured Schwann Cells

**Figure 5:**
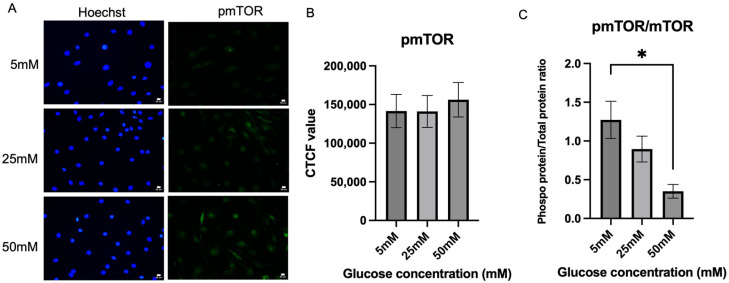
Expression of phosphorylated mTOR. (**A**) Representative immunofluorescence micrographs of Schwann cells expressing phosphorylated mTOR under 40× magnification. Scale bar = 50 µm. Schwann cells have been cultured in 5 mM, 25 mM, and 50 mM of glucose for 5 days and stained for phosphorylated mTOR. (**B**) Corrected total cell fluorescence for phosphorylated mTOR in three different culture media (5 mM, 25 mM, 50 mM). The fluorescence intensity of phosphorylated mTOR was quantified to observe the effect of hyperglycemia on the phosphorylated mTOR activity. (**C**) Quantification of pmTOR/mTOR ratio. The mean CTCF value was presented as mean ± SD. * *p* < 0.01, (*n* = 3, three independent experiments).

Following mTOR expression, phosphorylated mTOR was also evaluated in Schwann cells in the hyperglycemic condition. Figure 5A shows the fluorescence intensity of expression of p-mTOR by Schwann cells in the three different glucose concentration media. Figure 5B shows that there was no significant difference in the p-mTOR expression level between the three different glucose media. However, a slight increase in the p-mTOR expression level was observed in the 50 mM glucose media, which is the hyperglycemic condition with a mean of (156,208 ± 22,361), when compared to 25 mM glucose concentration (141,144 ± 20,658), an increase of 10.67%, *p* = 0.8764, and 5 mM glucose concentration (141,558 ± 21,547), *p* = 0.8826. Figure 5C shows the ratio of pmTOR/mTOR; the mean values for the 5 mm, 25 mm, and 50 mm groups were 1.273 ± 0.2402, 0.8960 ± 0.1666, and 0.3510 ± 0.0883, respectively. A statistically significant difference was observed between the 5 mm and 50 mm groups (*p* = 0.0179), indicating that the mTOR activation is inhibited as glucose concentration increases.

## 3. Discussion

Diabetic peripheral neuropathy (DPN) is a complex disorder where Schwann cell dysfunction, or Schwannopathy, plays a pivotal role in the progression of the disease [22]. Emerging evidence suggests that Schwann cell dysfunction may occur independently of axonal damage, contributing significantly to the pathology of DPN [23]. Research suggests that hyperglycemia affects Schwann cells, and in severe cases, it can cause demyelination, leading to central and peripheral sensitization in diabetic patients [24]. In line with that, we studied the plasticity of Schwann cells under high glucose conditions. This study is the first to investigate the regulation of the mTOR pathway and its interaction with key transcription factors such as c-Jun, Krox-20, p75^NTR^, MBP, and AKR1B1 in Schwann cells under hyperglycemic conditions. Our study hypothesized that hyperglycemia might influence Schwann cell responses through the regulation of the mTOR pathway.

In the context of peripheral nerve injury, Schwann cells reprogram into a repair phenotype to support axonal regeneration during Wallerian degeneration, with c-Jun being selectively upregulated [18]. In our study, c-Jun expression showed a non-significant upward trend at 50 mM glucose compared to 5 mM glucose. These observations are consistent with previous studies, which reported an increase in c-Jun expression following ascorbic acid-induced peripheral nerve injury [25]. Similarly, an in vitro model of Wallerian degeneration also showed elevated c-Jun expression, indicating the reprogramming of nerves into a repair phenotype [26]. Moreover, the upregulation of c-Jun can also occur without physical nerve injury, as seen in hereditary demyelinating neuropathies, where it helps prevent the excessive secondary loss of sensory axons [27]. The absence of statistical significance in our data indicates that this finding should be regarded as a preliminary observation. Further studies with larger sample sizes are needed to confirm whether c-Jun is truly involved in Schwann cell adaptation under hyperglycemia.

On the other hand, Krox-20 expression showed a downward trend at 50 mM glucose, although this change was not statistically significant. During nerve injury, Krox-20 is downregulated to facilitate Schwann cell dedifferentiation and repair [28], and the microenvironment can further inhibit Krox-20 expression, preventing satellite glial cells from adopting a Schwann cell-like phenotype [29]. In our study, the non-significant decrease in Krox-20 may suggest a potential similarity to these injury-related changes.

Notably, clinical studies in diabetic patients support this speculation, as sural nerve biopsies have revealed Schwann cell dedifferentiation with reduced myelin proteins and increased p75^NTR^ expression, along with ultrastructural changes such as segmental demyelination and axonal degeneration, closely resembling the Wallerian-like response observed after peripheral nerve injury [30,31]. Regarding p75^NTR^, it is well established that this receptor is expressed during Schwann cell development and in response to peripheral nerve injury [32,33]. In our study, p75^NTR^ expression showed an upward but non-significant trend under high-glucose conditions. While this aligns with reports linking p75^NTR^ to an immature/repair phenotype and protective roles in neuropathy, the lack of statistical significance means our finding should be considered preliminary. The elevated c-Jun expression in hyperglycemic environments may explain the concurrent rise in p75^NTR^ levels, as activation of c-Jun has been shown to regulate the expression of neurotrophin receptors, including p75^NTR^, during peripheral nerve injury [34]. Further, p75^NTR^ expression in Schwann cells is often associated with an immature/repair phenotype and may serve a protective role, as deficiency of this receptor has been shown to exacerbate diabetic neuropathy through immune pathway overactivation and lysosomal stress, with related miRNAs further linking p75^NTR^ signaling to autophagy and mTOR regulation [35].

Conversely, MBP expression significantly declined with increasing glucose levels, indicating a reduction in the myelinating phenotype under hyperglycemic stress. These results are consistent with a previous study that reported a reduction in MBP, MAG, and P0, alongside the upregulation of p75^NTR^, which has been linked to Schwann cell dedifferentiation in both in vivo and in vitro models, potentially due to sorbitol accumulation from polyol pathway hyperactivation, although the exact mechanism remains unclear [36]. Likely, a significant reduction in MBP was also reported in the oligodendrocytes that were exposed to high glucose [37]. This reduction in expression plays a crucial role in how cells respond to nerve injury, enabling Schwann cells to aid in axonal repair and regeneration [7,28]. Taken together, our significant findings highlight that hyperglycemia induces myelin protein loss through MBP downregulation, while other observed changes (such as p75^NTR^ increase) remain non-significant trends requiring further validation. Collectively, these changes indicate that hyperglycemia may promote a shift in Schwann cell differentiation status. The upward trends in c-Jun and p75^NTR^, both established markers of the repair phenotype, together with the significant reduction in MBP and the downward trend in Krox-20, markers of the myelinating phenotype, suggest that Schwann cells under high-glucose stress begin to reprogram away from a myelinating state toward features of a repair-like phenotype. This pattern resembles the dedifferentiation process observed during Wallerian degeneration and peripheral nerve injury, where Schwann cells adopt an immature/repair phenotype to facilitate axonal survival and regeneration. Although some changes did not reach statistical significance, the overall expression profile supports the interpretation that hyperglycemia primes Schwann cells for a reparative response.

This study also examined the effects of hyperglycemia on the expression of aldose reductase (AR), also known as AKR1B1, which serves as a key indicator of polyol pathway activation. Overactivation of AR has been linked to the accumulation of sorbitol and fructose, promoting osmotic stress, oxidative imbalance, and pro-inflammatory responses in neural tissues [38,39]. More recently, modulation of AR has been shown to attenuate microglial metabolic reprogramming and neuropathic pain in diabetic models, highlighting its functional relevance across neural and glial contexts [40]. In our study, AR expression showed only a slight, non-significant increase in Schwann cells cultured under hyperglycemic conditions, consistent with previous reports that primary Schwann cell cultures are not substantially affected by moderate hyperglycemia (20–30 mM) but respond more strongly to severe hyperosmotic stress (≥100 mM [41]. The AR is not substantially affected by moderate hyperglycemic condition (20–30 mM), but the response to severe hyperosmotic stress (≥100 mM) is due to the differences in the Schwann cell line used. The isolated primary Schwann cell culture did not increase the level of aldose reductase compared to control (5.6 mM) in the period of incubation to high glucose for 7 days, and no sorbitol accumulation was observed. However, the treatment with an AR inhibitor such as SNK-860 (fidarestat), reduced the sorbitol intracellular level in high glucose, showing that glucose is converted to sorbitol by aldose reductase. When the hyperosmotic stress elicited by the addition of sodium chloride (NaCl) or raffinose to the medium was applied, the AR activity in isolated primary Schwann cells increased [41]. This is due to the hyperosmotic conditions that can induce AR mRNA and increase AR activity in cultured cells of various origins [42,43,44,45]. In contrast, in the IMS32 cell, which is the spontaneously immortalized cell established from adult ICR mice, a significant increase in AR mRNA/protein expression was observed under exposure to high glucose (≥30 mM) conditions. However, the reason behind the increase of the polyol pathway in IMS32, but not in primary cultured Schwann cells cultured in high glucose concentration (20–30 mM), has not yet been discovered [20].

We recognize that while 72 h exposure to 25–50 mM glucose was sufficient to induce Schwann cell reprogramming markers (such as c-Jun upregulation and MBP downregulation) and to reveal alterations in mTOR phosphorylation, more prolonged exposure or more severe hyperglycemic stress may be required to fully activate apoptotic cascades or elicit stronger effects on downstream mTORC1 targets. For example, Zhu et al. (2018) reported that 50 mM glucose for 72 h inhibited the mTORC1/S6K1 pathway and induced apoptosis in Schwann cells [46], suggesting that higher doses or longer exposure may amplify these effects. Future studies will therefore examine extended incubation periods and higher glucose concentrations to determine whether the paradoxical mTOR profile observed here reflects an early adaptive response or progresses toward sustained pathway inhibition.

Our observation of increased total mTOR with a concomitant reduction in p-mTOR suggests a feedback-inhibited state in Schwann cells rather than simple pathway activation. Previous work has demonstrated that high glucose can modulate other signaling cascades in Schwann cells, such as the ERK pathway, which was shown to be activated and to influence cell viability and differentiation [47]. In addition, hyperglycemia-induced apoptosis of Schwann cells has been linked to inhibition of mTORC1/S6K1 signaling, supporting the concept that glucose stress suppresses anabolic pathways despite changes in protein abundance [46]. Interestingly, Nunes et al. (2021) reported that mitochondrial dysfunction in Schwann cells activates mTORC1 and c-Jun, as evidenced by increased phosphorylation of downstream targets S6 and 4E-BP1, despite no significant change in mTOR Ser2448 phosphorylation [16]. In contrast, our findings under hyperglycemic stress reveal increased total mTOR but reduced Ser2448 phosphorylation, suggesting a distinct feedback-inhibited state. This divergence highlights how different cellular stressors, metabolic versus mitochondrial, can differentially modulate the mTOR pathway in Schwann cells. Furthermore, oxidative and ER stress are known to induce REDD1 and activate AMPK, which in turn repress Rheb–mTORC1 signaling via TSC2, providing another mechanism for the uncoupling between mTOR expression and phosphorylation [48]. Taken together, these studies suggest that the “mTOR paradox” observed here reflects a convergence of stress-driven negative feedback and impaired Akt signaling, rather than uniform pathway activation. Future studies will therefore assess Akt phosphorylation (Thr308, Ser473), downstream mTORC1 readouts (p-S6K1 (Thr389), p-4EBP1 (Thr37/46), ULK1 (Ser757)), and regulators such as REDD1 and sestrin2 in Schwann cells exposed to high glucose, with pharmacological interventions (rapamycin, Torin-1, SC79, NAC/apocynin) to clarify the mechanistic basis of this paradox in diabetic neuropathy.

Similarly, Hei et al. demonstrated that hyperglycemia can exacerbate neuronal injury by activating components of the mTOR pathway under both normoxic and ischemic conditions [49]. Chronic mTOR activation by sustained high glucose may trigger compensatory negative feedback mechanisms that reduce its phosphorylation over time, even when total mTOR protein levels remain elevated. This could involve increased oxidative stress, depletion of upstream activators, or upregulation of endogenous mTOR inhibitors [50]. Moreover, mTOR is reported to be overactivated in obesity and insulin resistance models, which elevated serine phosphorylation of IRS-1, leading to impaired insulin signaling to Akt [51]. These findings are further supported by studies showing that mitochondrial damage in Schwann cells under diabetic conditions can lead to upregulation of both c-Jun and mTOR components without necessarily altering their phosphorylation status [16]. We propose that these apparent discrepancies reflect the tissue- and context-specific roles of mTOR signaling. In systemic metabolic disorders such as obesity and insulin resistance, chronic nutrient overload results in persistent mTORC1 overactivation, driving IRS-1 serine phosphorylation and impairing Akt signaling. By contrast, in Schwann cells, hyperglycemia and mitochondrial stress appear to induce a feedback-inhibited state, in which total mTOR and c-Jun levels are elevated but phosphorylation is not, possibly as an adaptive mechanism to limit excessive anabolic signaling under stress [16,52].

Additionally, our data showed a slight but non-significant upward trend in aldose reductase (AR) expression under high glucose conditions. While this may point to potential polyol pathway involvement, the finding does not reach statistical significance and should be considered preliminary. The polyol pathway has been reported to stimulate NADPH oxidase (Nox), a protein kinase C-dependent enzyme. Notably, hyperglycemia has been shown to increase Nox1 expression, which is typically low under physiological conditions, and Nox1 has been implicated in mTOR activation and peripheral nervous system injury in diabetic models. Hyperactivation of the polyol pathway consumes NADPH and generates excess NADH, shifting the cellular redox balance toward oxidative stress. This can enhance Nox enzyme activity, particularly Nox1 and Nox4, leading to superoxide production and contributing to Schwann cell dysfunction in diabetes [53]. In our study, however, the lack of statistical significance for AR expression indicates that its role remains inconclusive, and further studies with larger sample sizes are necessary to confirm whether polyol pathway–Nox interactions contribute to Schwann cell stress under hyperglycemia.

## 4. Limitations and Future Perspectives

This study has several limitations that should be acknowledged. First, the experimental work was conducted exclusively in vitro using primary Schwann cells, which may not fully replicate peripheral nerves’ complex in vivo environment under diabetic conditions. This controlled setting did not account for factors such as immune cell interactions, vascular changes, and systemic metabolic influences. Future studies should therefore incorporate in vivo validation using diabetic animal models, such as streptozotocin-induced diabetic mice, to confirm whether Schwann cell reprogramming and the associated changes in c-Jun, Krox-20, MBP, p75^NTR^, mTOR, and AKR1B1 expression also occur in the context of diabetic neuropathy. Protein expression can then be evaluated directly from Schwann cells isolated from diabetic nerves, providing stronger translational relevance to the findings.

Second, while the study identified changes in the expression levels of key markers such as c-Jun, Krox-20, MBP, p75^NTR^, and mTOR, it did not investigate the upstream signaling pathways or downstream functional outcomes of these molecular changes. It will be essential in future work to examine whether the observed molecular reprogramming translates into measurable changes in nerve function, for example, by assessing nerve conduction velocity, axonal regeneration capacity, or behavioral outcomes in diabetic neuropathy models. This functional validation would help establish whether the molecular signatures identified here are directly linked to impaired or adaptive nerve repair processes. Third, the study was limited by the relatively small sample size, which may have reduced the statistical power to detect more subtle but biologically relevant changes. Future studies with larger sample numbers will be needed to confirm whether these trends reflect true biological differences. Fourth, the ability of Schwann cells to sustain a repair phenotype can also be reduced by the loss of axonal contact. While axonal deprivation initially induces c-Jun upregulation and repair cell reprogramming, prolonged absence of regenerating axons results in a decline of c-Jun expression within 8–10 weeks, thereby limiting Schwann cell repair capacity [54,55,56]. In diabetic neuropathy, additional factors such as chronic hyperglycemia, oxidative stress, vascular insufficiency, and inflammation may further compromise Schwann cell regenerative potential, highlighting the importance of in vivo models that capture these influences. Fifth, this study did not assess gene expression profiles, which could provide deeper insights into the transcriptional regulation underlying Schwann cell reprogramming. Future studies will explore these aspects, including gene expression analysis, in vivo validation, and functional assays, to better understand how Schwann cells respond to high glucose and how this affects nerve repair.

## 5. Materials and Methods

### 5.1. Cell Isolation and Culture

Isolation of primary Schwann cells was conducted following ethical guidelines approved by the Animal Ethics Committee of the Institute of Medical Science Technology, Universiti Kuala Lumpur, under approval number AEC/MESTECH-UNIKL/2018/001. Wistar rats, aged 9 to 12 weeks, were euthanized, sterilized with 70% alcohol, and handled by ethical standards. Under sterile conditions, the sciatic nerves were carefully dissected, with the surrounding tissues and epineurium removed. The nerves were then cut into 1 mm segments and digested enzymatically with 0.05% collagenase-A for 90 min at 37 °C. After digestion, the resulting material was filtered through a sterile 40 µm cell strainer, and the cell pellet was collected and resuspended in Schwann cell isolation media. DMEM D-valine (Sigma Aldrich, Saint Louis, MO, USA Cat. No. D9443) was supplemented with 10% Fetal Bovine Serum (FBS; Gibco, Thornton, NSW, Australia, Cat. No. A4766801), 1% GlutaMAX (Sigma Aldrich, Saint Louis, MO, USA, Cat. No. G7513), 1% Penicillin-Streptomycin (Sigma Aldrich, Saint Louis, MO, USA, Cat. No. P0781), 0.5% Amphotericin B (Gibco, Waltham, MA, USA, Cat. No. 15290026), 1% N-2 Supplement, 100× (Gibco, Waltham, MA, USA 02451, Cat. No. 17502048), 20 µg/mL Bovine Pituitary Extract (BPE; Sigma Aldrich, Saint Louis, MO, USA, Cat. No. P1476), and 5 µM Forskolin (Sigma Aldrich, Saint Louis, MO, USA, Cat. No. F6886). Schwann cells were seeded onto plates precoated with Poly-L-lysine (Sigma Aldrich, Saint Louis, MO, USA, Cat. No. P4707) and Laminin (Sigma Aldrich, Saint Louis, MO, USA, Cat. No. L4544) and cultured for 12 weeks at 37 °C with 5% CO_2_ until reaching ~80% confluency.

### 5.2. Immunocytochemical Labeling

Cells were fixed with 4% paraformaldehyde (in PBS) and permeabilized with 0.1% Triton X-100. Then, cells were blocked with 3% bovine serum albumin (BSA) in PBS for 60 min at room temperature. Then, the samples were incubated at 4 °C overnight with primary antibodies and washed three times with PBS before incubating with fluorescent-tagged secondary antibodies for 90 min at room temperature. The primary antibodies used were anti-Krox20 rabbit (1:1000, Invitrogen, Carlsbad, CA, USA, Cat. No. PA5-112409), polyclonal anti-c-Jun mouse (1:200, Santa Cruz, Dallas, TX, USA, Cat.No.sc-166540), phospho-mTOR-S2448 Rabbit (1:200, ABclonal, Cat. No. AP0094), mTOR Rabbit (1:100, ABclonal, Woburn, MA, USA, Cat. No. A11354), AKR1B1 Rabbit (1:200, ABclonal, Woburn, MA, USA, Cat. No. A1684), anti-MBP mouse monoclonal (Santa Cruz, Dallas, TX, USA, Cat. No. sc-271524) (1:200), and anti-p75^NTR^ (Promega, Madison, WI, USA, Cat. No. G3231) (1:200). The secondary antibodies used were DyLight 549-conjugated anti-mouse IgG (1:100, Vector Laboratories, Newark, CA, USA, Cat. No. DI-2549) and goat anti-rabbit IgG fluorescein (1:100, Vector Laboratories, Newark, CA, USA, Cat. No. F1-1000). The primary antibodies were diluted in 1% BSA solution, while the secondary antibodies were diluted in 1% BSA solution containing 1% goat and 1% horse serum. Lastly, Hoechst 33342 (Thermo Fisher Scientific, Waltham, MA, USA, Cat. No. 62249) (1:200) was added to stain for nuclei for 30 min at room temperature and washed three times with PBS before imaging. Cells were imaged using a Nikon Eclipse Ni (Nikon Corporation, Tokyo, Japan) fluorescence microscope equipped with Lumenera’s Infinity 3 digital microscopy camera (Lumenera Corporation, Ottawa, ON, Canada).

### 5.3. Quantitative Analysis on the Expression of the Biomarkers Using Corrected Total Cell Fluorescence (CTCF) Measurement

Quantitative analysis of biomarker expression was performed by measuring Corrected Total Cell Fluorescence (CTCF) using ImageJ software (ImageJ 1.54g), as previously described [57]. For each image, individual cells were outlined as regions of interest (ROIs), and the integrated density, area, and mean background fluorescence intensity were obtained. Background intensity was determined from three randomly selected areas lacking fluorescence in each image to normalize against autofluorescence. The CTCF was calculated using the following formula: CTCF = Integrated Density − (Area of selected cell × Mean Background Intensity). A total of three images were analyzed per group, with 30–40 cells measured, and the resulting values were exported to Microsoft Excel (Microsoft 365 Apps for Enterprise) for quantification of fluorescence intensity.

### 5.4. Statistical Analysis

GraphPad Prism (Version 9.0.0) was used for all statistical analyses. The Shapiro–Wilk test was preferred for determining normality due to the small sample size. For data that were not normally distributed, log transformation was applied to achieve normality, allowing for parametric tests to be conducted. All data were expressed as mean ± S.D from three independent experiments. Statistical comparisons were performed by using one-way ANOVA, with statistical significance set at *p* < 0.05.

## 6. Conclusions

This study provides new insights into the molecular mechanisms by which hyperglycemia affects Schwann cells in the context of diabetic peripheral neuropathy (DPN). Our findings demonstrate that high glucose conditions (50 mM) lead to a significant reduction in MBP expression, indicating a marked loss of the myelinating phenotype and initiation of Schwann cell dedifferentiation under hyperglycemic stress. This change was accompanied by significant upregulation of total mTOR expression and a concurrent reduction in phosphorylated mTOR (Ser2448), suggesting dysregulated mTOR signaling. In addition, trends of increased c-Jun and p75^NTR^ expression alongside decreased Krox-20 point to a reprogramming toward a repair-like phenotype, a process inherently linked to demyelination and impaired nerve support. Taken together, these results highlight impaired myelin protein expression, repair-associated phenotypic changes, and altered mTOR pathway activity as central features of Schwann cell responses to hyperglycemia that may contribute to the development of neuropathy.

## Data Availability

The data presented in this study are available on request from the corresponding author.
